# Liquid AP-UV-MALDI Enables Stable Ion Yields of Multiply Charged Peptide and Protein Ions for Sensitive Analysis by Mass Spectrometry**

**DOI:** 10.1002/anie.201208628

**Published:** 2013-01-22

**Authors:** Rainer Cramer, Alexander Pirkl, Franz Hillenkamp, Klaus Dreisewerd

**Affiliations:** Institute for Hygiene, University of MünsterRobert-Koch-Strasse 41, 48149 Münster (Germany); Institute for Medical Physics and Biophysics, University of MünsterRobert-Koch-Strasse 31, 48149 Münster (Germany)

**Keywords:** AP-MALDI, liquid matrices, mass spectrometry, multiply charged peptides

In biological mass spectrometry (MS), two ionization techniques are predominantly employed for the analysis of larger biomolecules, such as polypeptides. These are nano-electrospray ionization^1^,^2^ (nanoESI) and matrix-assisted laser desorption/ionization^3^,^4^ (MALDI). Both techniques are considered to be “soft”, allowing the desorption and ionization of intact molecular analyte species and thus their successful mass-spectrometric analysis. One of the main differences between these two ionization techniques lies in their ability to produce multiply charged ions. MALDI typically generates singly charged peptide ions whereas nanoESI easily provides multiply charged ions, even for peptides as low as 1000 Da in mass. The production of highly charged ions is desirable as this allows the use of mass analyzers, such as ion traps (including orbitraps) and hybrid quadrupole instruments, which typically offer only a limited *m*/*z* range (<2000–4000). It also enables more informative fragmentation spectra using techniques such as collision-induced dissociation (CID) and electron capture/transfer dissociation (ECD/ETD) in combination with tandem MS (MS/MS).^5^,^6^ Thus, there is a clear advantage of using ESI in research areas where peptide sequencing, or in general, the structural elucidation of biomolecules by MS/MS is required. Nonetheless, MALDI with its higher tolerance to contaminants and additives, ease-of-operation, potential for high-speed and automated sample preparation and analysis as well as its MS imaging capabilities makes it an ionization technique that can cover bioanalytical areas for which ESI is less suitable.^7^,^8^ If these strengths could be combined with the analytical power of multiply charged ions, new instrumental configurations and large-scale proteomic analyses based on MALDI MS(/MS) would become feasible.

In previous papers, the benefits of liquid matrices in IR- and UV-MALDI MS were demonstrated.^9^–^11^ For liquid UV-MALDI MS, these benefits include a stable and durable analyte ion yield over thousands of laser shots and the capacity of these matrices to accommodate matrix additives that can change the properties of the MALDI sample significantly. It has been shown that these properties of liquid matrices can be exploited for highly accurate analyte quantitation^11^ and a wide coverage of MALDI sample pH values.^12^ The broad pH range enabled tryptic digestion within the MALDI sample and the detection of its products by MS.^12^

Atmospheric pressure (AP)-MALDI has been shown to facilitate the formation of multiply charged protein as well as peptide ions, although the sensitivity is lower and an infrared laser must be employed.^13^ Doubly charged peptide ions were also recorded under intermediate pressure in UV-MALDI MS and under vacuum pressure using IR-MALDI MS,^14^ but with a much lower signal intensity than for singly charged ions. The possibility of generating multiply charged ions with higher yield by changing the matrix and ion-source design was discussed.^14^ Subsequently, Zenobi et al. reported alternative MALDI sample-preparation methods to increase the yield of multiply charged ions. One of these employed electrospray deposition of at least 200 pmol of analyte on various preformed MALDI matrix layers, showing that under specific conditions highly charged insulin ions can be detected albeit at a low signal-to-noise ratio.^15^

Herein, we report progress in achieving high and prolonged yields of multiply charged peptide and protein ions using liquid UV-MALDI matrices and an AP ion source with an ion transfer tube that can be used at variable elevated temperatures of up to 400 °C. The liquid matrices described herein are based on 2,5-dihydroxybenzoic acid (DHB) or α-cyano-4-hydroxycinnamic acid (CHCA) with the addition of glycerol and triethylamine in various concentrations. The employed laser optics and ion-source design, together with a measured maximum laser energy of around 10 μJ on target allowed an achievable maximum fluence of under 2000 J m^−2^. Compared to laserspray ionization (LSI), a recently introduced approach for laser-induced generation of multiply charged ions, this value is more than one order of magnitude below the reported LSI fluence range of 40–150 kJ m^−2^.^16^,^17^ A major disadvantage of these LSI irradiation conditions is the typically rapid depletion of the sample. However, in the present study multiply charged ions were obtained with laser energies as low as around 1 μJ, resulting in a fluence of under 200 J m^−2^, which is within the range of typical UV-MALDI MS fluences^18^ and more than two orders of magnitude below the reported LSI fluence range. Thus, continuous analyte ion signal detection from tens of thousands of consecutive laser shots was achieved with concomitant low sample consumption.

To date, analytes in the mid-femtomole range were sufficient to produce predominantly multiply rather than singly charged ions with a stable analyte ion beam for up to 36 000 laser shots, that is, for a one-hour data acquisition. Ion charge states varied depending on the exact nature of the liquid MALDI matrix, and alkali cationization decreased with charge state while sizable matrix adduct ion formation was only observed for singly charged ions.

Figure 1 shows a liquid UV-AP-MALDI mass spectrum of MK-bradykinin (sequence: MKRPPGFSPFR) revealing singly, doubly, and triply charged analyte ions. In general, adduct ion formation was far less for the multiply charged ions than for singly charged ions. As seen in Figure 1, there were no significant adduct ions detected for the triply charged MK-bradykinin ion, while significant amounts of analyte/cation clusters with alkali metals and the matrix chromophore compounds were detected for the singly charged ion species. The absence of adduct ions for multiply charged ions is an important observation since liquid MALDI samples are typically a good source of salt cations and thus generally support cation adduct formation.

**Figure 1:**
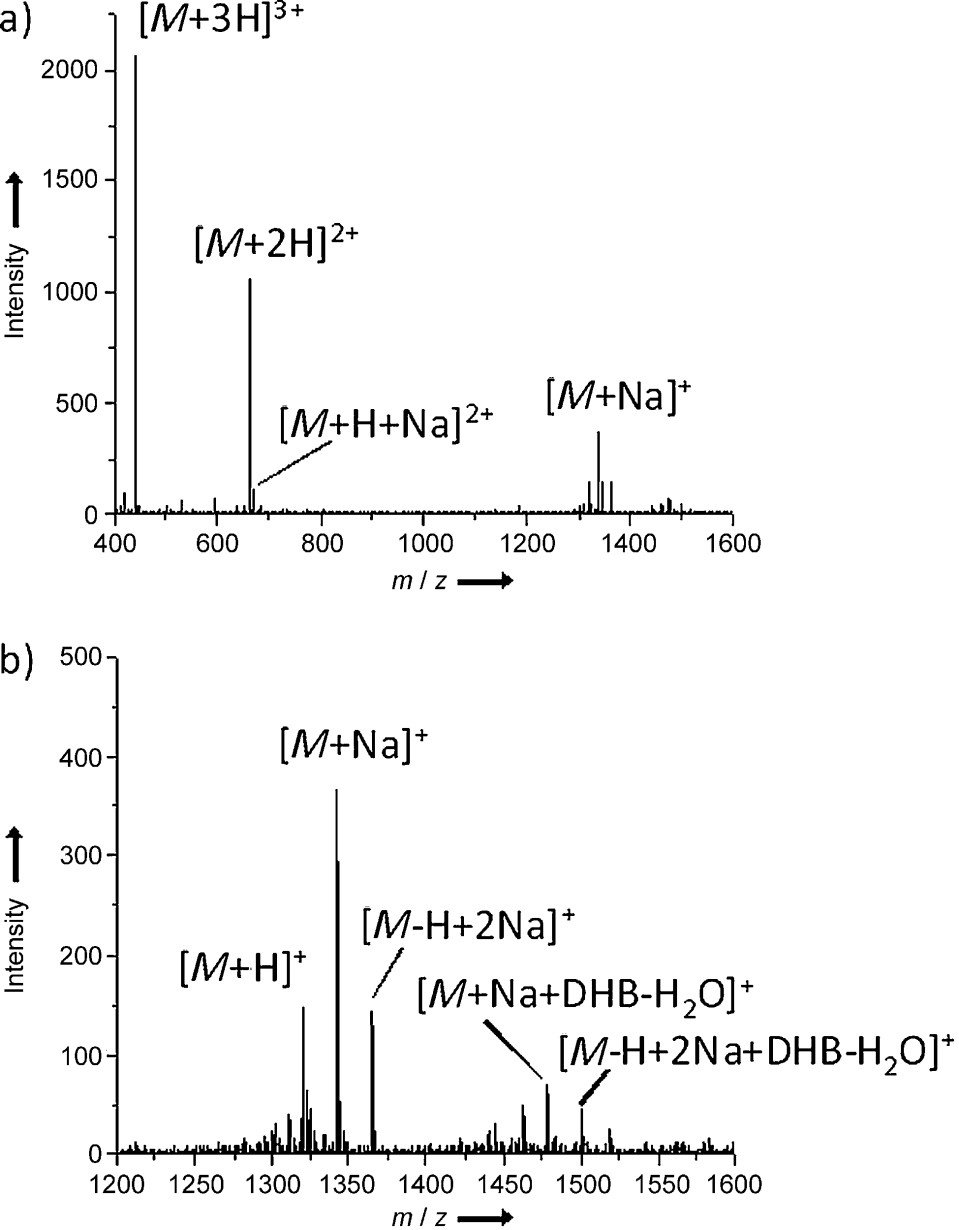
AP-UV-MALDI mass spectrum of MK-bradykinin (sequence: MKRPPGFSPFR), showing a) the *m*/*z* range 400–1600 and b) the *m*/*z* range 1200–1600. The matrix was the DHB-based liquid matrix containing approximately 20 % glycerol before volatile solvent evaporation.

As expected, the generation of multiply charged peptide ions greatly facilitated their fragmentation and thus sequencing. Figure 2 displays the CID MS/MS fragment spectra of MK-bradykinin for the doubly and triply charged ion species. In general, these fragmentation spectra, displaying mainly b- and y-type ion series and other associated fragment ions as well as iminium ions, are similar to CID MS/MS spectra of doubly and triply charged peptide ions generated by other soft ionization techniques.

**Figure 2:**
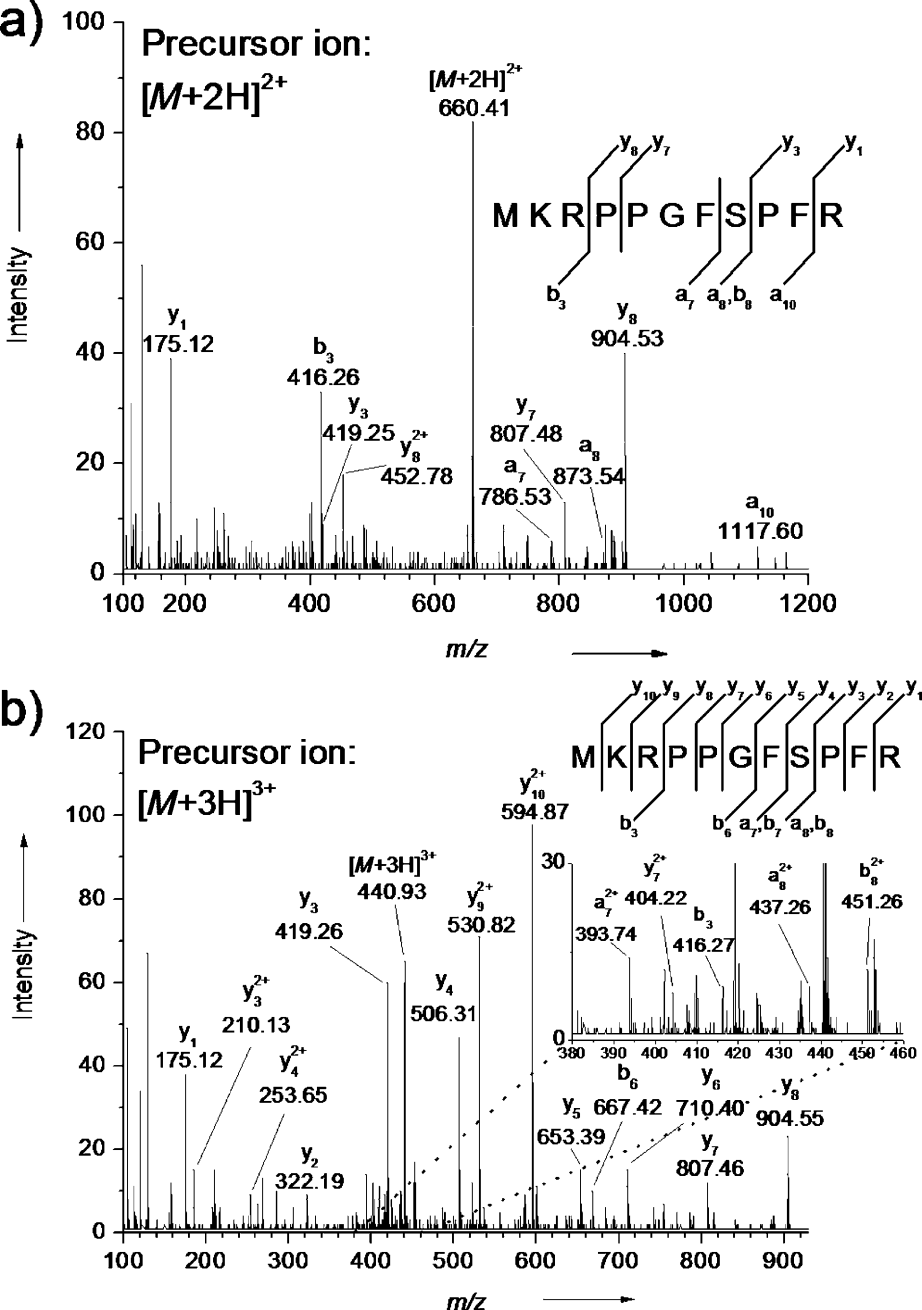
AP-UV-MALDI CID MS/MS spectra of the a) doubly and b) triply protonated MK-bradykinin ions. The matrix was the DHB-based liquid matrix containing approximately 20 % glycerol before the evaporation of volatile solvents. The CID collision potentials were 35 V (a) and 20 V (b).

Although the formation of multiply charged ions was highly favored, it was possible to detect significant amounts of singly charged ions for lower mass analytes, similar to other ionization techniques, such as ESI. However, it should be emphasized that in all investigated cases using liquid matrices it was easier to detect the analyte as a multiply rather than singly charged ion species. This is also evident in the results from two optimization experiments. The first was undertaken to optimize the transfer-tube temperature to obtain the highest yields of multiply charged analyte ions (Supporting Information, Figure S1). Apart from finding an optimum temperature of 200–250 °C for the 1-3-5-10 liquid matrix (see Experimental Section), the data also suggest that the formation of singly charged ions is far less affected by the temperature than the formation of multiply charged species. This pattern can also be seen in the second investigation, determining the optimum amount of glycerol in the liquid matrix (Supporting Information, Figure S2 and S3). In this case, the signal of the singly charged ion species actually decreases when yields of the multiply charged increase. For all the liquid matrices investigated, glycerol (or better, the liquidity of the sample) was an essential component for the formation of multiply charged ions. Typically, a sufficient amount of glycerol that guaranteed a fully liquid MALDI sample appeared to be best. Adding further amounts of glycerol eventually led to lower multiply charged ion signals (data not shown), presumably a result of a decreased optical absorption and an analyte dilution effect, while any lower amount led to (partial) crystallization of the solid matrix components, which was concomitant with a decrease in the detection of multiply charged ions.

Changing the composition of the liquid matrices appears to have a dramatic effect on the detectable charge states and their distribution. For melittin, the 1-1-10 matrix (see Experimental Section) enables just the detection of the doubly charged ion species whereas switching to the DHB-based liquid matrix (see Experimental Section) facilitates the detection of the triply and quadruply charged ions with a negligible signal for the doubly charged species (Supporting Information, Figure S2 and S3). The potential of the DHB-based liquid matrix to generate higher charge states was also observed for MK-bradykinin.

One of the advantages of liquid MALDI samples is the relatively stable ion flux and spot durability during laser ablation, which are a result of the self-healing properties of the liquid. Figure 3 shows the liquid (DHB-based matrix) MALDI MS spectrum and total ion chromatogram (TIC) of 1800 scans (30 min acquisition) of a melittin sample. The extracted ion chromatogram (EIC) for the [*M*+4 H]^4+^ melittin ion shows a similarly stable ion yield, and spectra generated by combining only the scans from the first minute were virtually identical to the combination of the scans in the last minute (Supporting Information, Figure S4). In this case, 500 fmol of melittin and a laser-pulse repetition rate of 10 Hz was employed, that is, 18 000 shots for the entire acquisition with an average analyte consumption of less than 30 amol per laser shot. Random sampling of individual scans throughout the acquisition showed that each scan (1 s; 10 laser shots) had a sufficient analyte signal-to-noise ratio for unambiguous detection of the multiply charged ions.

**Figure 3:**
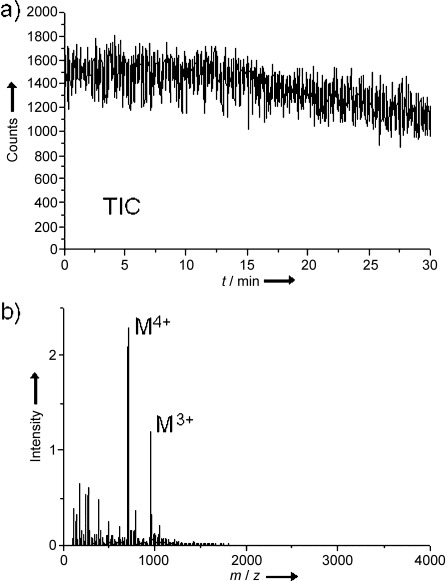
a) Total ion chromatogram (TIC) over a 30 min data acquisition using a liquid MALDI sample containing 500 fmol melittin. b) AP-UV-MALDI mass spectrum of the sum of all scans of the above acquisition. The matrix was the DHB-based liquid matrix containing approximately 20 % glycerol before evaporation of volatile solvents, and the laser repetition rate was 10 Hz.

To date, the lowest amount of analyte successfully analyzed was 50 fmol of melittin, prepared on the target plate (Supporting Information, Figure S5). Other analytes tested were insulin and myoglobin (Figure 4). The observed charge state distributions from these three analytes appear to be very narrow. Thus, the generation of specific charge states and their distributions as a result of the choice of matrix seems to be very flexible and somewhat different than ESI. However, some of the effects are putatively attributable to changes in pH value, solvents, or surfactants as well as extraction voltage and transfer-tube temperature, all of which are similar to effects described for ESI.^19^–^22^

**Figure 4:**
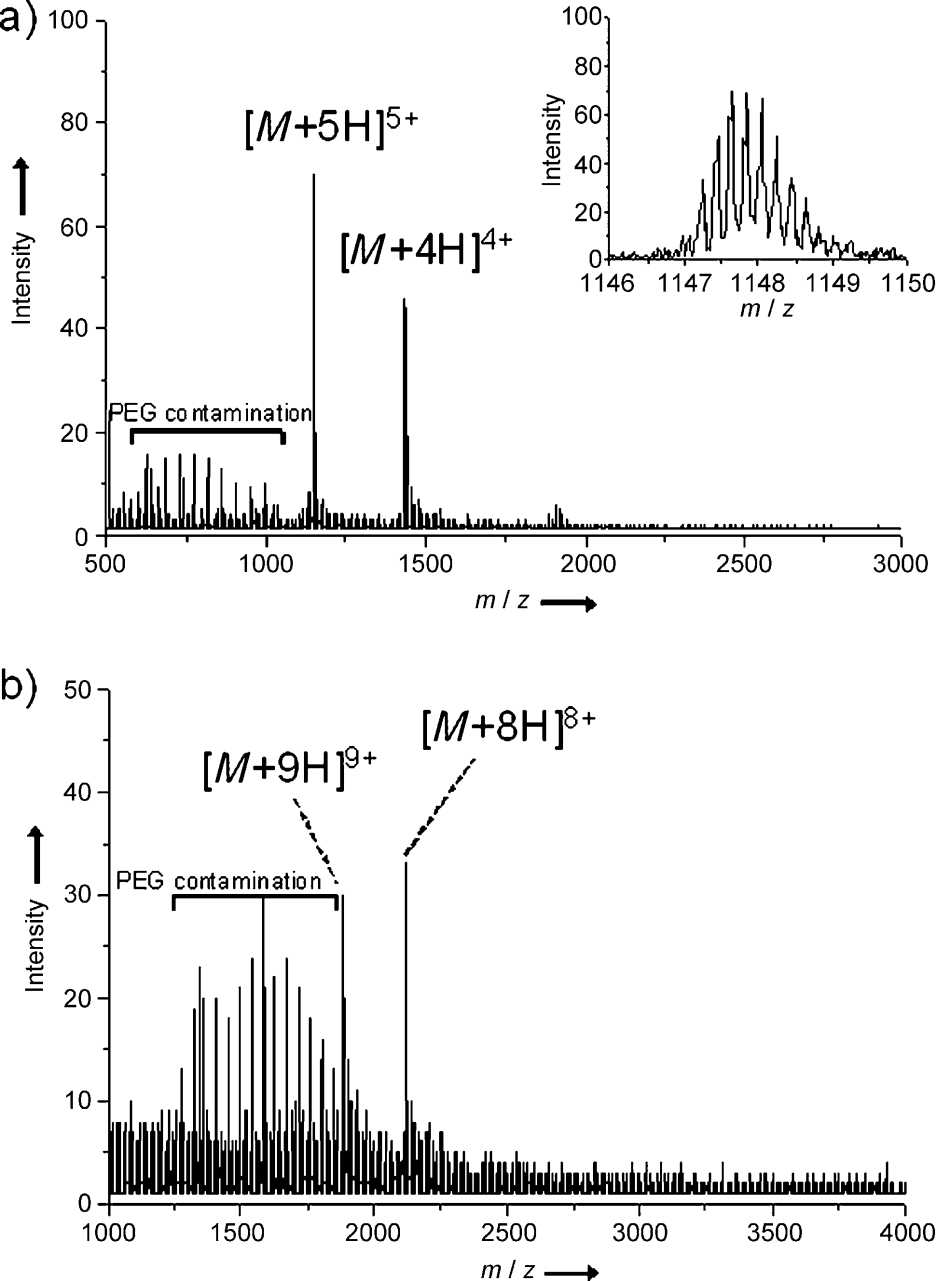
a) AP-UV-MALDI mass spectrum of 5 pmol porcine insulin. The matrix was the DHB-based liquid matrix containing approximately 15 % glycerol before evaporation of volatile solvents. b) AP-UV-MALDI mass spectrum of 5 pmol horse heart myoglobin. The matrix was the CHCA-based 1-1-10 liquid matrix containing approximately 30 % glycerol before evaporation of volatile solvents. The pH value was above 7, as determined by a pH paper test strip. The detected [*M*+8 H]^8+^ and [*M*+9 H]^9+^ analyte ions can be attributed to the apo-form of myoglobin. In both spectra various polyethylene glycol (PEG) contaminant ions are also visible (possibly because of adverse storage conditions of the two samples over two years as fully diluted analyte solutions in plastic tubes).

## Experimental Section

The first step in the preparation of the MALDI matrices was the addition of 20–100 mm ammonium phosphate/methanol (1:1; v:v) to the solid UV matrix compound 2,5-dihydroxybenzoic acid (DHB) or α-cyano-4-hydroxycinnamic acid (CHCA) in a ratio of 10:1 (v[μL]:w[mg]). For DHB-based liquid matrices, glycerol was added and the resultant mixture was thoroughly vortexed and then sonicated for 5–10 min. For the CHCA-based liquid matrix 1-1-10, triethylamine was added at a tenth of the volume of the ammonium phosphate/methanol solvent and vortexed with subsequent addition of various volumes of glycerol, whereas the CHCA-based 1-3-5-10 matrix was prepared by specifically adding triethylamine using 30 % of the volume of the ammonium phosphate/methanol solvent and another addition of glycerol at 50 % volume. After each addition, the mixture was thoroughly vortexed and then sonicated for 5–10 min. Peptides and proteins were dissolved in water at concentrations of 10^−7^–10^−3^ m. MALDI samples were prepared directly on the stainless steel target plate by spotting 0.5–1 μL of the analyte solution first and subsequently 0.5–1 μL of the matrix solution. The samples were left at ambient conditions for 15–30 min to allow evaporation of the volatile solvent components.

Mass spectra were acquired on a modified Q-Star Pulsar i instrument (AB Sciex, Toronto, Canada) with a custom-made AP-MALDI source based on a design previously reported.^23^ Details of the source can be found in the Supporting Information. Unless stated otherwise, mass spectra were recorded at a transfer-tube temperature of 225 °C by accumulating about 60 scans with a scan time of 1 s.

## Supplementary material

As a service to our authors and readers, this journal provides supporting information supplied by the authors. Such materials are peer reviewed and may be re-organized for online delivery, but are not copy-edited or typeset. Technical support issues arising from supporting information (other than missing files) should be addressed to the authors.

## References

[1] Körner R, Wilm M, Morand K, Schubert M, Mann M (1996). J. Am. Soc. Mass Spectrom.

[2] Wilm M, Shevchenko A, Houthaeve T, Breit S, Schweigerer L, Fotsis T, Mann M (1996). Nature.

[3] Karas M, Bachmann D, Bahr U, Hillenkamp F (1987). Int. J. Mass Spectrom. Ion Proc.

[4] Karas M, Hillenkamp F (1988). Anal. Chem.

[5] Glish GL, Burinsky DJ (2008). J. Am. Soc. Mass Spectrom.

[6] Wiesner J, Premsler T, Sickmann A (2008). Proteomics.

[7] Drancourt M (2010). Clin. Microbiol. Infect.

[8] Seeley EH, Schwamborn K, Caprioli RM (2011). J. Biol. Chem.

[9] Cramer R, Burlingame AL (2000). Rapid Commun. Mass Spectrom.

[10] Cramer R, Corless S (2005). Proteomics.

[11] Palmblad M, Cramer R (2007). J. Am. Soc. Mass Spectrom.

[12] Towers M, Cramer R (2007). Spectroscopy.

[13] König S, Kollas O, Dreisewerd K (2007). Anal. Chem.

[14] Cramer R, Corless S (2001). Rapid Commun. Mass Spectrom.

[15] Kononikhin AS, Nikolaev EN, Frankevich V, Zenobi R (2005). Eur. J. Mass Spectrom.

[16] Inutan ED, Wager-Miller J, Mackie K, Trimpin S (2012). Anal. Chem.

[17] Trimpin S, Wang B, Inutan ED, Li J, Lietz CB, Harron A, Pagnottis VS, Sardelis D, McEwen CN (2012). J. Am. Soc. Mass Spectrom.

[18] Rohlfing A, Leisner A, Hillenkamp F, Dreisewerd K (2010). J. Phys. Chem. C.

[19] Konermann L, Douglas DJ (1998). Rapid Commun. Mass Spectrom.

[20] Loo RRO, Dales N, Andrews PC (1994). Protein Sci.

[21] Sterling HJ, Cassou CA, Susa AC, Williams ER (2012). Anal. Chem.

[22] Sterling HJ, Daly MP, Feld GK, Thoren KL, Kintzer AF, Krantz BA, Williams ER (2010). J. Am. Soc. Mass Spectrom.

[23] Schneider BB, Lock C, Covey TR (2005). J. Am. Soc. Mass Spectrom.

