# Supplementary material for: Liquid AP-UV-MALDI Enables Stable Ion Yields of Multiply Charged Peptide and Protein Ions for Sensitive Analysis by Mass Spectrometry
Source: Angew Chem Int Ed Engl. 2013 Jan 22;52(8):2364–7. doi: 10.1002/anie.201208628 (PMC3592991; doi:10.1002/anie.201208628)
Supplement: Supplementary file 1 [file anie0052-2364-SD1.pdf]

Angewandte Chemie
Eine Zeitschrift der Gesellschaft Deutscher Chemiker

Supporting Information

© Wiley-VCH 2013

69451 Weinheim, Germany

Liquid AP-UV-MALDI Enables Stable Ion Yields of Multiply Charged Peptide and Protein Ions for Sensitive Analysis by Mass Spectrometry**

Rainer Cramer, Alexander Pirkl, Franz Hillenkamp, and Klaus Dreisewerd*

anie_201208628_sm_miscellaneous_information.pdf

Experimental

Materials. All components of the MALDI matrices were purchased from Sigma-Aldrich (Germany). The solvents were of HPLC-grade. MK-bradykinin, melittin, porcine insulin and horse heart myoglobin were obtained from Sigma-Aldrich. All analyte solutions were made up in HPLC-grade water. The MALDI target was a home-made stainless steel plate.

Mass Spectrometry. Mass spectra were acquired on a modified Q-Star Pulsar i instrument (AB Sciex, Toronto, Canada) with a custom-made AP-MALDI source based on a design previously reported (Reference 23 and Figure S6). This design was further modified by adding a heat coil (B08-00200, 90W; GC-heat, Waldbroel, Germany) to the ion transfer tube, which could then be heated up to 400°C (measured on the inside wall of the transfer tube using a K-type thermocouple). The beam of a Nd:YAG laser (λ = 355 nm, τ = 10 ns; Diodescope, Bioptic, Berlin, Germany) was coupled into an optical fibre with a core diameter of 50 µm (CeramOptec, Bonn, Germany). The end surface of the fibre was imaged 1:1 onto the sample surface at an incidence angle of ~70° relative to the target plate normal (ion beam axis). Further details of the source can be found in Figure S6. In each experiment the distance between droplet surface and heated stainless steel transfer tube was adjusted to ~3 mm. The position of the laser beam and liquid sample film were adjusted such that ions were produced roughly on the ion beam axis (transfer tube axis). Unless stated otherwise, mass spectra were recorded at a transfer tube temperature of 225°C by accumulating ~60 scans with a scan time of 1 sec.

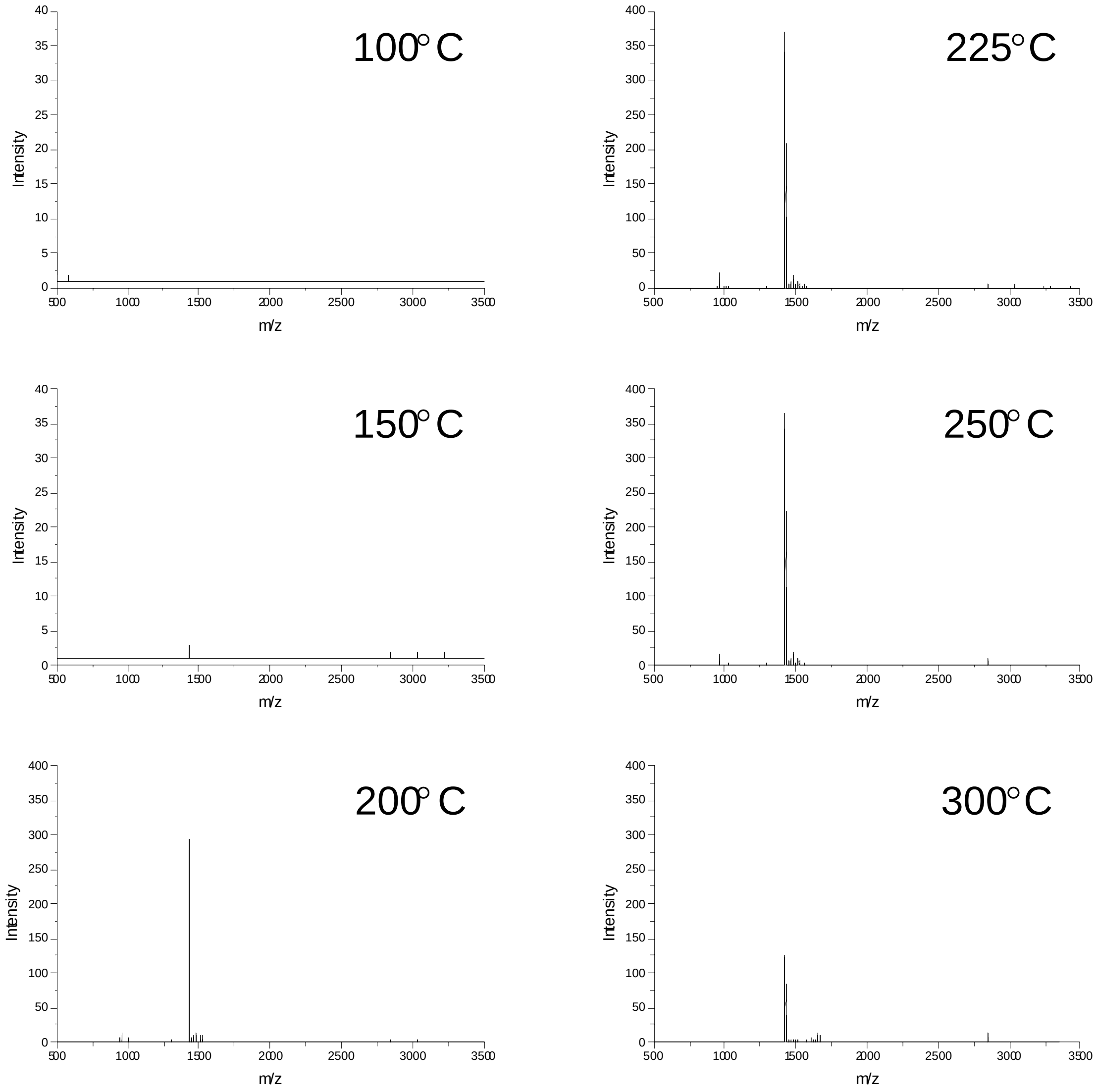

Figure S1:
AP-UV-MALDI mass spectra of melittin acquired at various transfer tube temperatures. The matrix was the CHCA-based 1-3-5-10 liquid matrix.

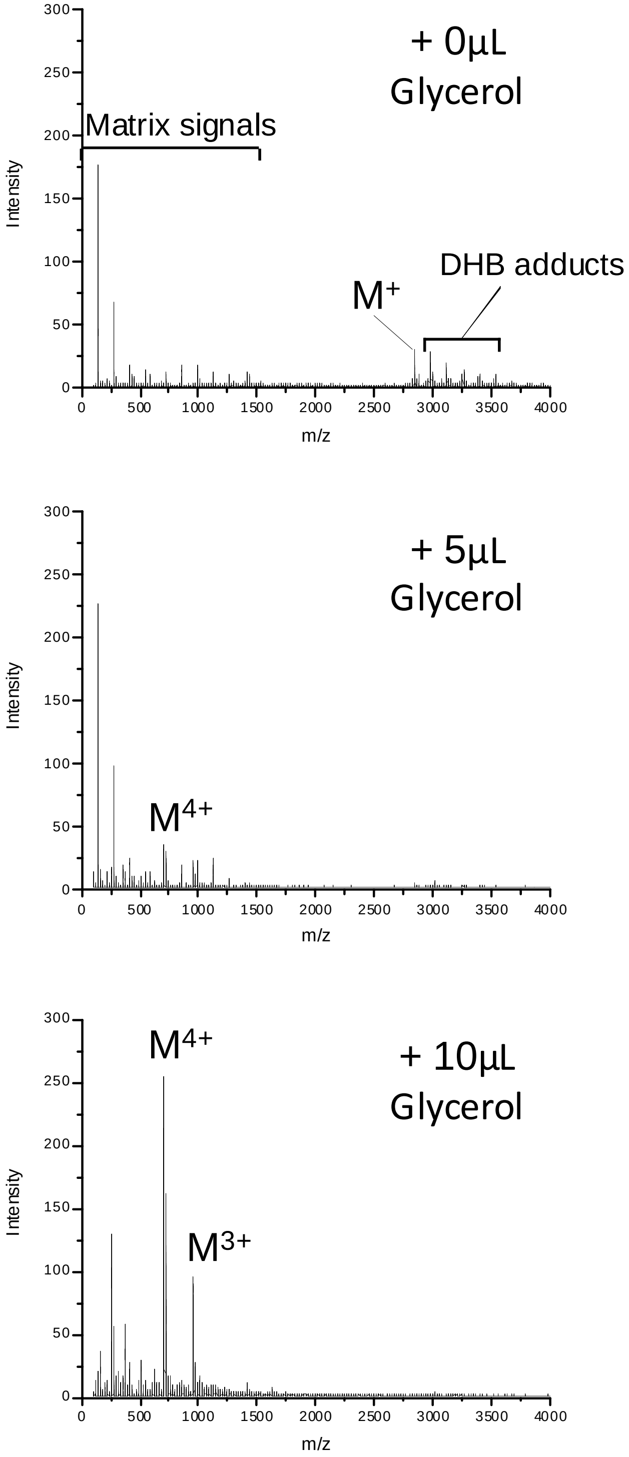

Figure S2:
AP-UV-MALDI mass spectra of melittin. The matrix was the DHB-based (liquid) matrix with the addition of 0, 5, and 10 µL of glycerol to 50 µL of matrix stock solution. The MALDI samples were prepared directly on the MALDI plate by spotting 0.5 µL of the analyte solution first and subsequently 0.5 µL of the matrix solution. For the sample preparation without any glycerol extensive DHB clusters were detected.

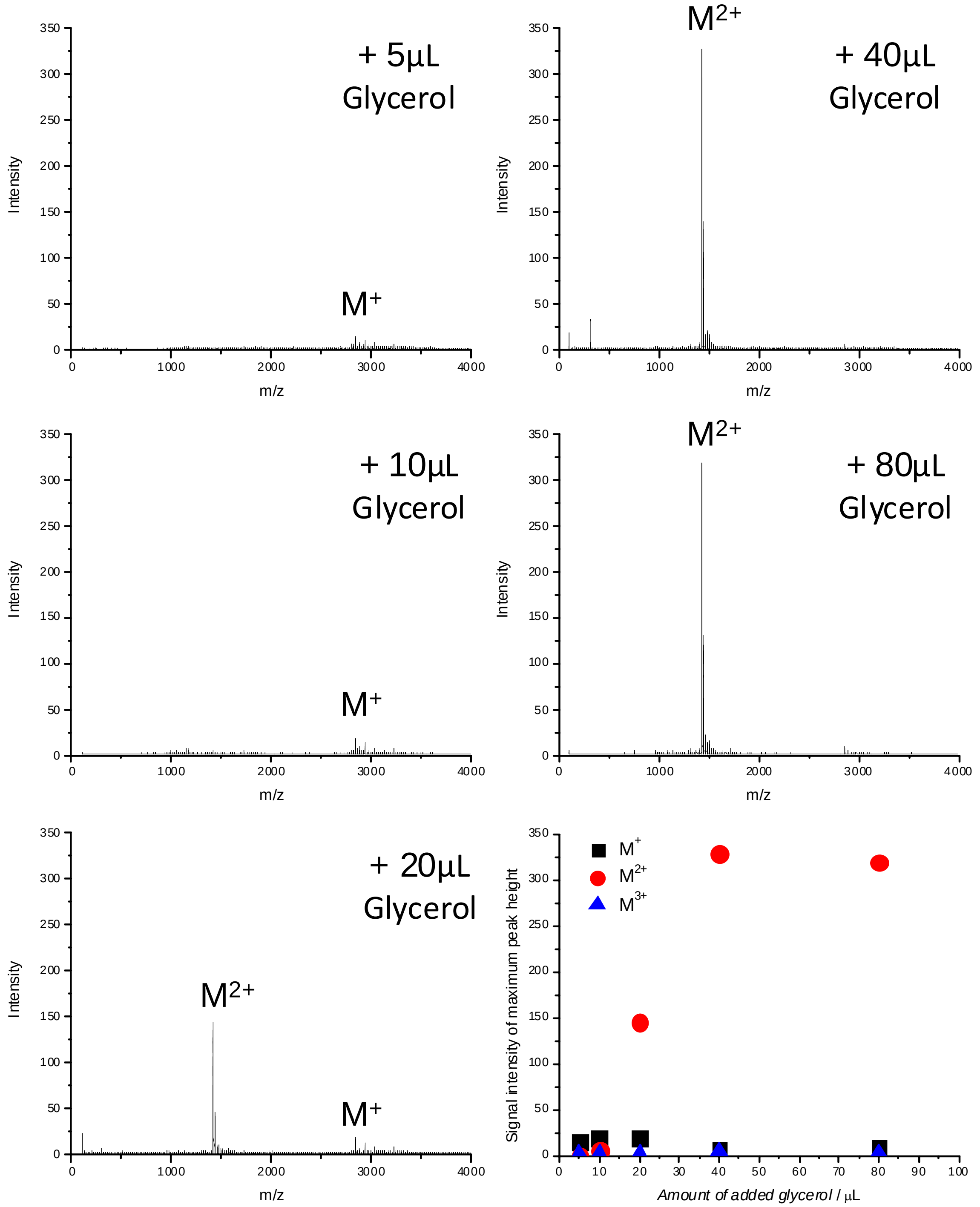

Figure S3:
AP-UV-MALDI mass spectra of melittin. The matrix was the CHCA-based 1-1-10 liquid matrix with the addition of 5, 10, 20, 40 and 80 µL of glycerol to 100 µL of matrix stock solution. The MALDI samples were prepared directly on the MALDI plate by spotting 0.5 µL of the analyte solution first and subsequently 0.5 µL of the matrix solution. The bottom right panel displays the signal intensities of the maximum peak height extracted from all five spectra for M^+, M^{2+}, and M^{3+}.

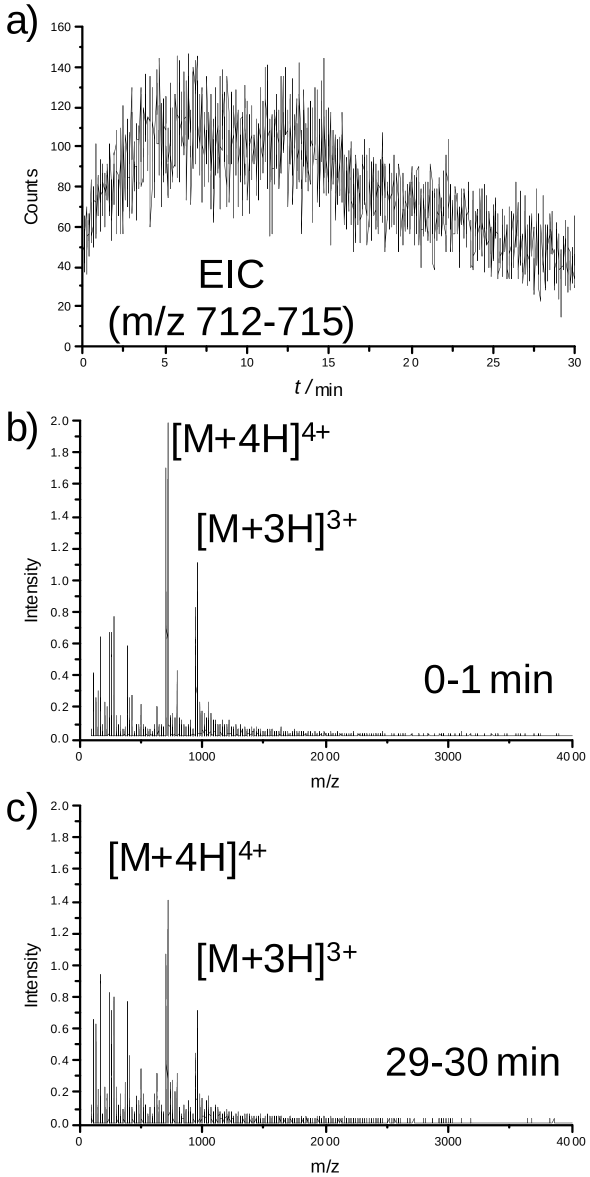

Figure S4:
a) Extracted ion chromatogram (EIC) with an m/z window of 712-715 over a 30-min acquisition of a liquid MALDI sample containing 500 fmol melittin (cf. Figure 3). AP-UV-MALDI mass spectra combining the scans of the b) first minute and c) last minute of the above acquisition. The matrix was the DHB-based liquid matrix containing ~20% glycerol before volatile solvent evaporation. The laser repetition rate was 10 Hz, and the transfer tube temperature was 225°C.

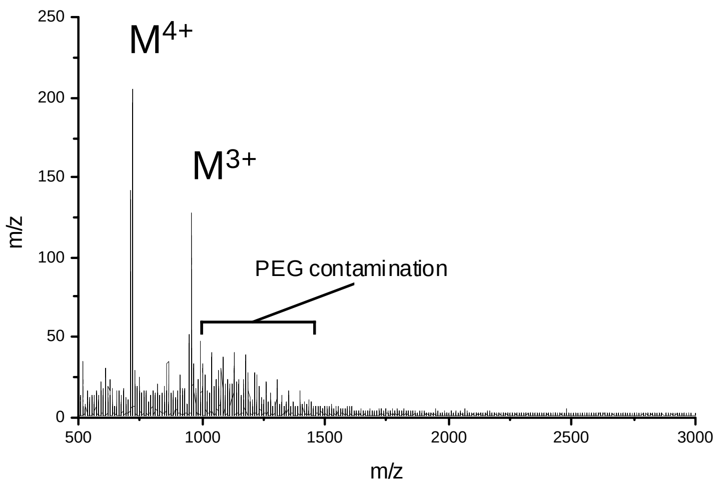

Figure S5:
AP-UV-MALDI mass spectrum of 50 fmol melittin. The matrix was the DHB-based liquid matrix containing ~20% glycerol before volatile solvent evaporation.

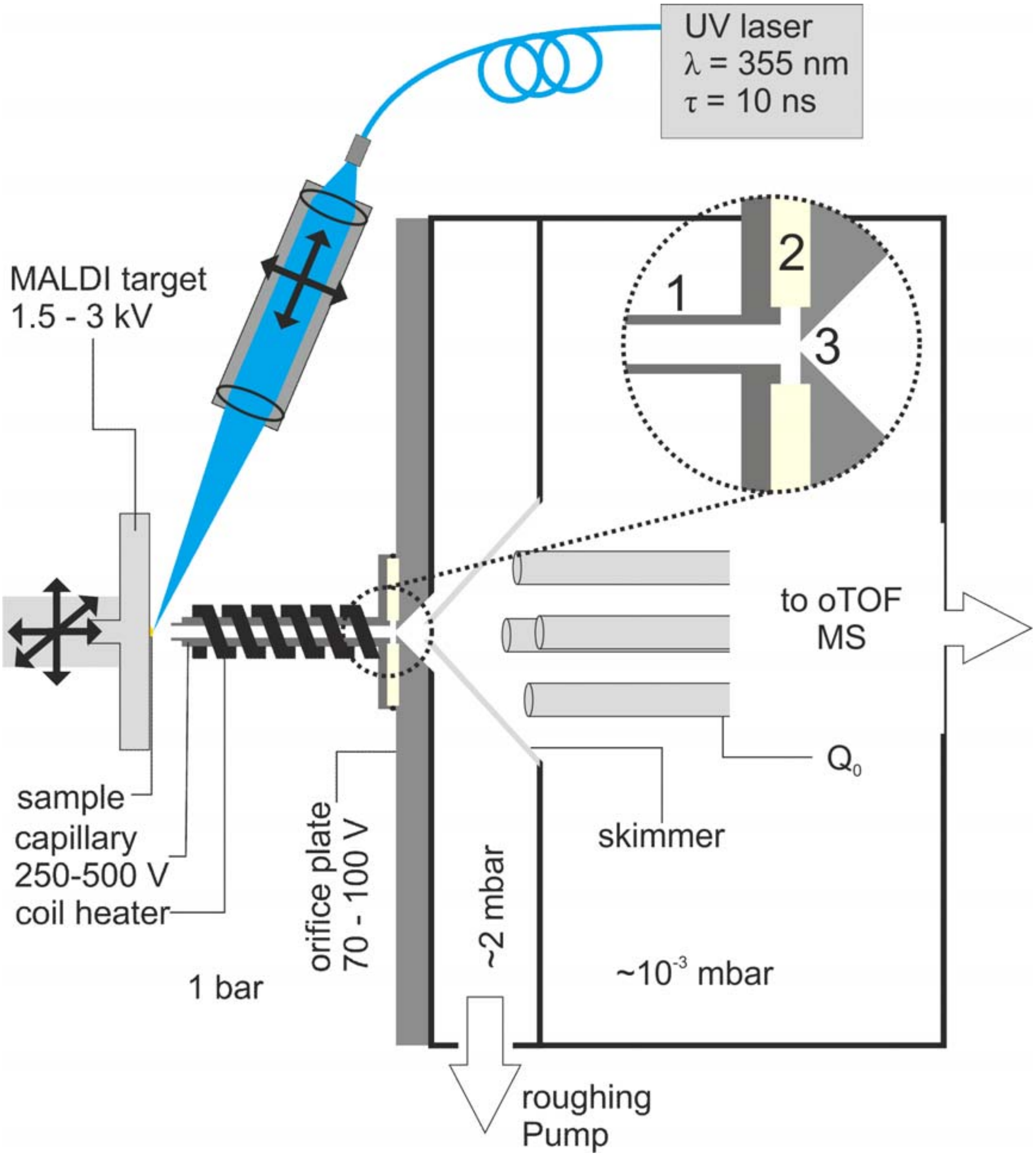

Figure S6:
Diagram of the ion source design used for all AP-UV-MALDI MS measurements. The inset shows a diagram of the particle discriminator interface adapted from reference 23. The heated transfer tube (1; $Ø_i$ = 2 mm, l = 40 mm) is separated from the standard ESI orifice plate (3) with its flow limiting orifice (Ø = 250 µm) by a ceramic spacer (2), producing a gap between ion transfer tube and orifice of 1-mm thickness and 4-mm diameter. Sealing is improved by an O-ring surrounding the ceramic spacer.

Materialien. Alle Komponenten der MALDI-Matrizes wurden von Sigma-Aldrich (Deutschland) erworben. Die Lösungsmittel waren von HPLC-Qualität. MK-Bradykinin, Melittin, Insulin vom Schwein und Myoglobin vom Pferd wurden ebenfalls von Sigma-Aldrich erworben. Alle Analytlösungen wurden mit HPLC-Wasser präpariert. Der MALDI-Probenteller war ein selbsthergestellten Edelstahlteller.

Massenspektrometrie. Massenspektren wurden an einem modifizierten Q-Star Pulsar i (AB Sciex, Toronto, Kanada) mit einer maßgeschneiderten AP-MALDI-Ionenquelle, die auf einem zuvor berichteten Design beruht,[23] aufgenommen (Reference 23 und Abbildung S6). Dieses Design wurde zusätzlich durch das Anbringen einer Heizspirale (GC-Heat, Waldbröl, Deutschland B08-00200, 90W) an das Ionentransferröhrchen ergänzt, welches dann bis 400°C (mit einem Typ-K-Thermoelement an der Innenwand des Ionentransferröhrchens gemessen) erhitzt werden konnte. Der Strahl eines frequenzverdreifachten Nd:YAG-Lasers (λ = 355 nm, τ = 10 ns; Diodescope, Bioptic, Berlin, Deutschland) wurde in eine optische Faser mit einem Kerndurchmesser von 50 µm (CeramOptec, Bonn, Deutschland) eingekoppelt. Die Endfläche der Faser wurde unter einem Einfallswinkel von ca. 70° bezüglich der Probentellernormalen (Ionenstrahlachse) 1:1 auf die Probenoberfläche abgebildet. Weitere Einzelheiten der Quelle sind in Abbildung S6 enthalten. Für jedes Experiment wurde der Abstand zwischen der Tröpfchenoberfläche und dem beheizten Edelstahl-Ionentransferröhrchen auf ~3 mm eingestellt. Die Positionen des Laserstrahls und des flüssigen Probenfilms wurden so eingestellt, daβ Ionen ungefähr auf der Ionenstrahlachse (Ionentransferröhrchenachse) erzeugt wurden. Sofern nicht anders angegeben, wurden die Massenspektren mit einer Temperatur des Ionentransferröhrchens von 225°C durch das Aufsummieren von ~60 *scans* mit einer *scan*-Zeit von 1 s aufgezeichnet.

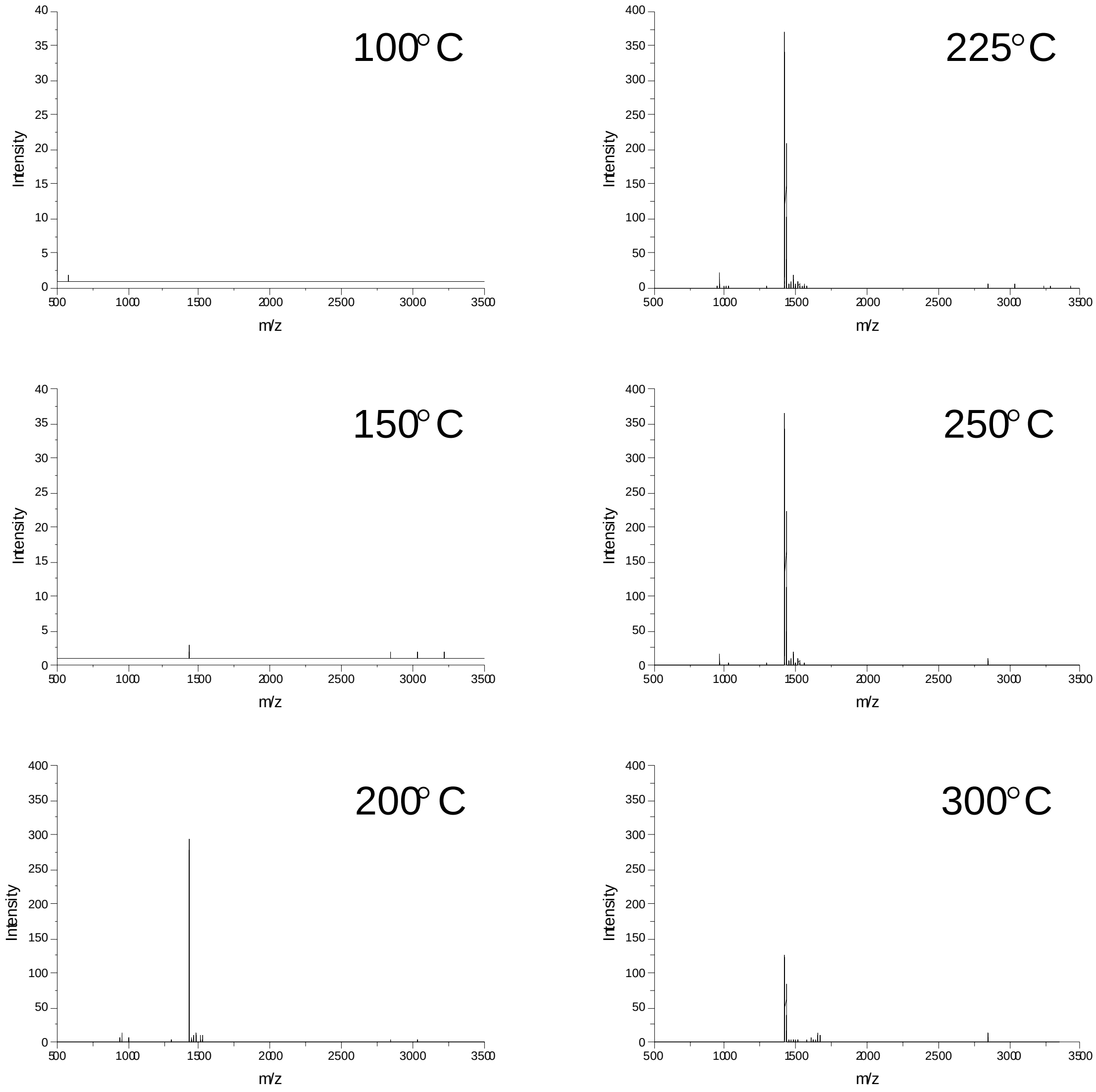

Abbildung S1:
AP-UV-MALDI-Massenspektrum von Melittin, aufgenommen bei verschiedenen Ionentransferröhrchen-Temperaturen. Matrix: CHCA-basierende 1-3-5-10 Flüssigmatrix.

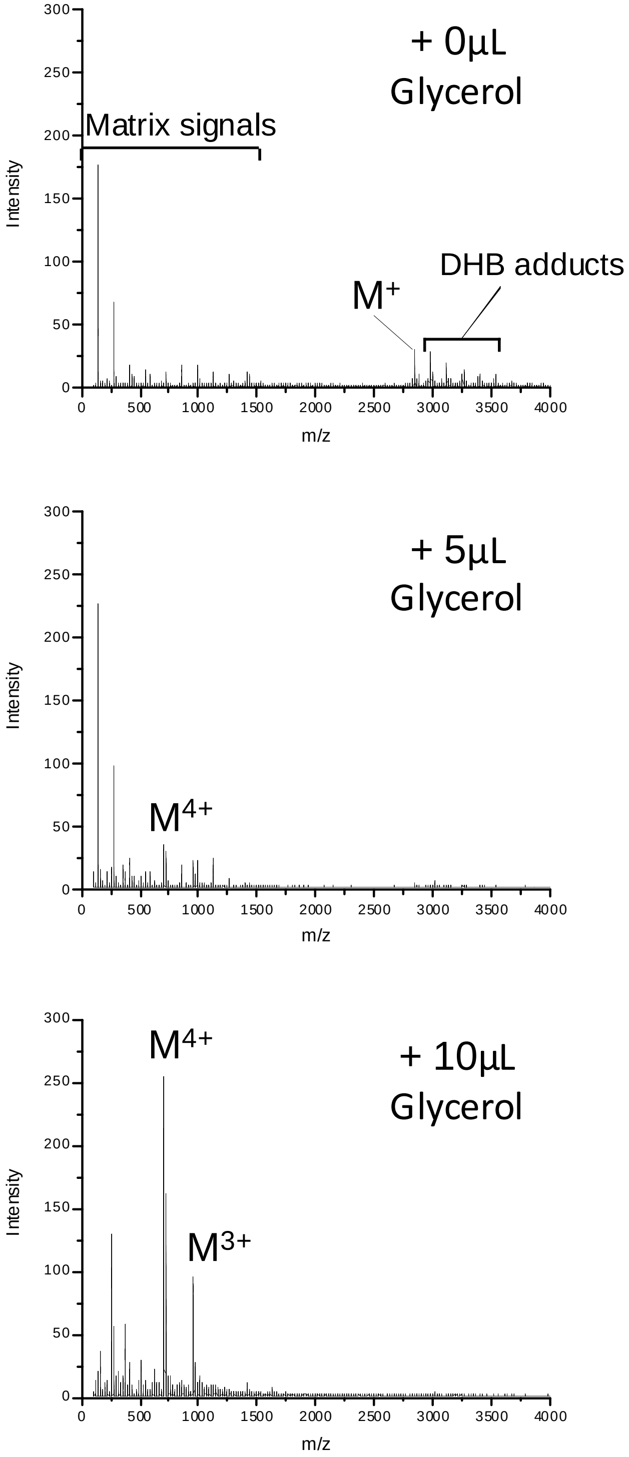

Abbildung S2:
AP-UV-MALDI-Massenspektrum von Melittin. Matrix: DHB-basierende Flüssigmatrix mit der Zugabe von 0, 5, und 10 µL Glycerin zu 50 µL Matrix-Stammlösung. Die MALDI-Proben wurden direkt auf dem Probenteller präpariert, in dem zuerst 0,5 µL Analytlösung und nachfolgend 0,5 µL Matrixlösung aufgetragen wurden. Bei der Probenpräparation ohne Glycerin wurden umfangreiche DHB-*Cluster* beobachtet.

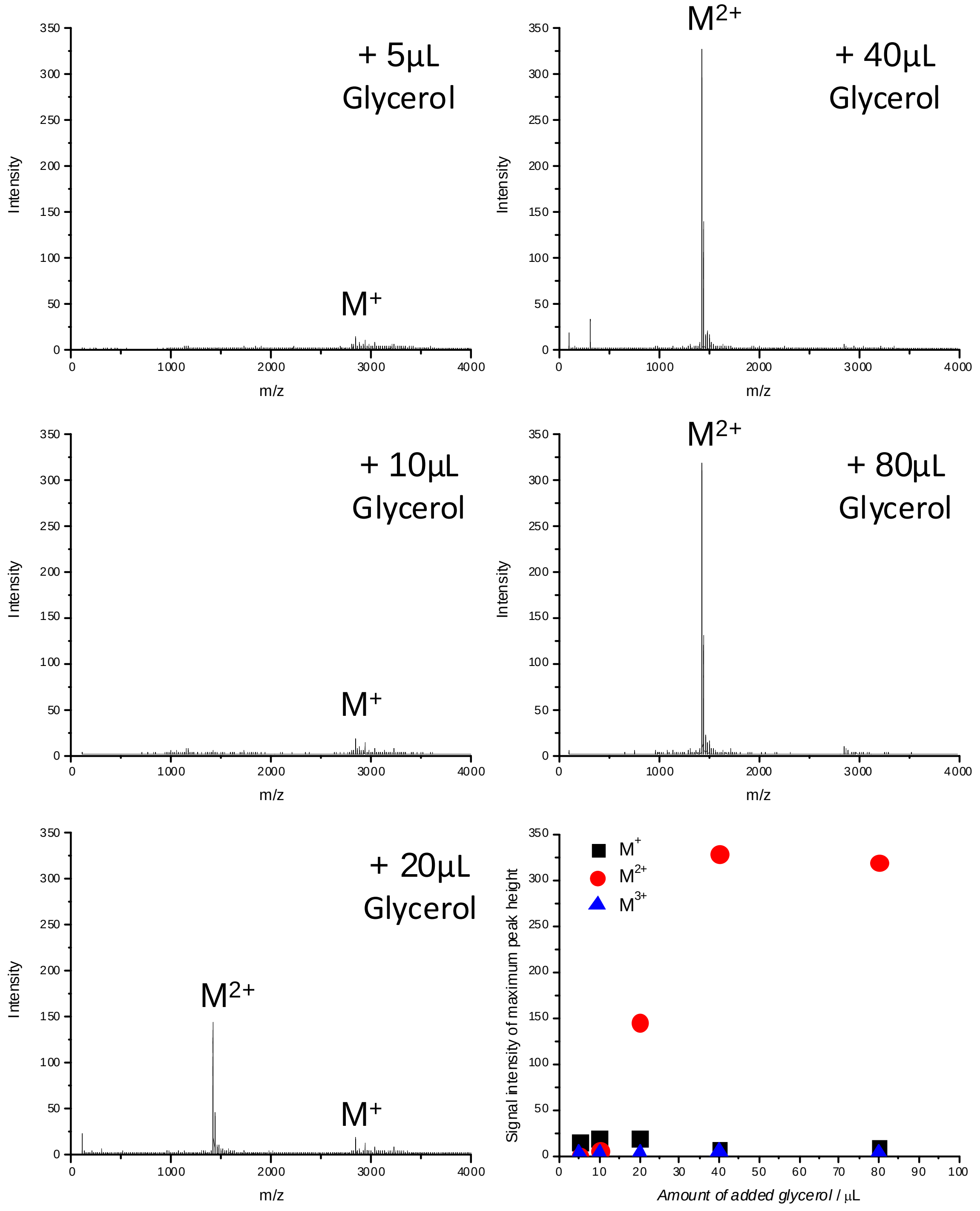

Abbildung S3:
AP-UV-MALDI-Massenspektrum von Melittin. Matrix: CHCA-basierende 1-1-10 Flüssigmatrix mit der Zugabe von 5, 10, 20, 40 und 80 µL Glycerin zu 100 µL Matrix-Stammlösung. Die MALDI-Proben wurden direkt auf dem Probenteller präpariert, in dem zuerst 0,5 µL Analytlösung und nachfolgend 0,5 µL Matrixlösung aufgetragen wurden. Der rechte untere Graph zeigt die Signalintensitäten der maximalen Peakhöhe für die M^{+}-, M^{2+}-, und M^{3+}-Ionen aus allen fünf Spektren.

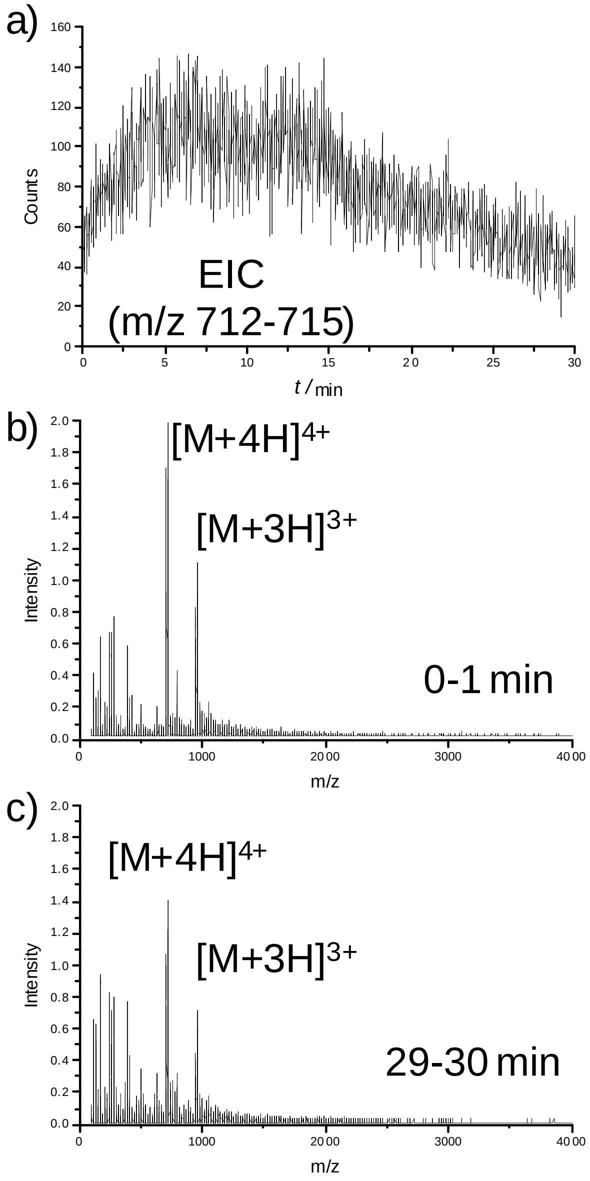

Abbildung S4:
a) *Extracted ion chromatogram* (EIC) mit einem m/z-Fenster von 712-715 über eine 30-minütige Datenaufnahme unter der Verwendung einer flüssigen Probe mit einer Gesamtmenge von 500 fmol Melittin (vgl. Abb. 3). AP-UV-MALDI-Massenspektren, erhalten aus der Summation der *scans* von der b) ersten Minute and c) letzten Minute der obigen Datenaufnahme. Matrix: DHB-basierende Flüssigmatrix mit ~20% Glycerin vor der Verdampfung volatiler Lösungsmittel. Laserpulsfrequenz: 10 Hz. Ionentransferröhrchen-Temperatur: 225°C.

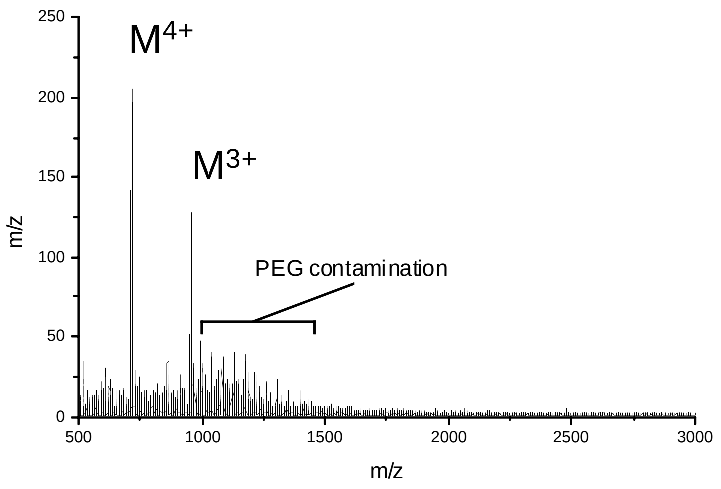

Abbildung S5:
AP-UV-MALDI-Massenspektrum von 50 fmol Melittin. Matrix: DHB-basierende Flüssigmatrix mit ~20% Glycerin vor der Verdampfung volatiler Lösungsmittel.

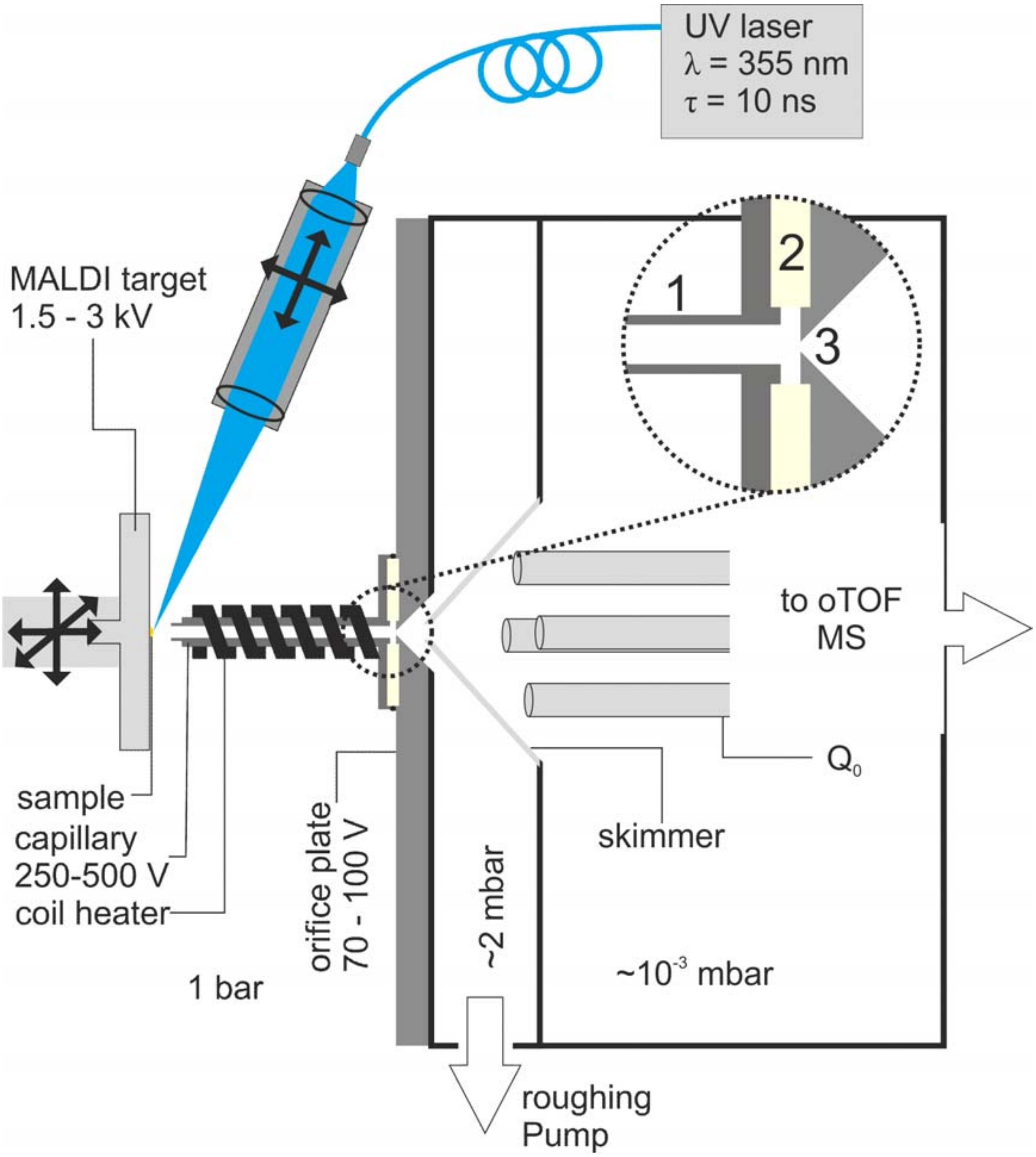

Abbildung S6:
Diagramm der Ionenquelle, die für alle AP-UV-MALDI-MS-Messungen verwendet wurde. Der Einschub zeigt eine schematische Darstellung des Teilchendiskriminators der Ionentransfereinheit, die nach Referenz 23 modifiziert wurde. Das geheizte Ionentransferröhrchen (1; $Ø_i$ = 2 mm, l = 40 mm) ist von der Standard-ESI-*orifice plate* (3) mit seiner Gasfluβ-limitierenden Öffnung (Ø = 250 µm) durch einen keramischen Abstandshalter (2) getrennt. Letzterer erzeugt einen Zwischenraum zwischen dem Ionentransferröhrchen und der *orifice plate* von 1 mm Dicke und 4 mm Durchmesser. Die Dichtigkeit wird durch einen O-Ring um den keramischen Abstandshalter verbessert.
